# Frailty still matters to health and survival in centenarians: the case of China

**DOI:** 10.1186/s12877-015-0159-0

**Published:** 2015-12-03

**Authors:** Danan Gu, Qiushi Feng

**Affiliations:** United Nations Population Division, Two UN Plaza, DC2-1910, New York, NY 10017 USA; Department of Sociology, National University of Singapore, Singapore, Singapore

**Keywords:** Centenarians, Frailty index, Cumulative deficit index, China, Mortality, Successful aging, Healthy aging, Healthy longevity, CLHLS

## Abstract

**Background:**

Frailty indicates accumulated vulnerability of adverse health outcomes in later life. Its robustness in predicting dependent living, falls, comorbidity, disability, health change, mortality, and health care utilization at older ages is well-documented. However, almost no studies have ever attempted to examine its robustness in centenarians, mainly due to data unavailability. This study examines prevalence of frailty in centenarians and its predictive powers on subsequent mortality and health conditions.

**Methods:**

We use a sample of 4434 centenarians from the 2002, 2005, 2008, and 2011 waves of the Chinese Longitudinal Healthy Longevity Survey (CLHLS), with elders in three younger age groups 65–79, 80–89, and 90–99 as comparisons. Frailty is measured by a cumulative deficit index (DI) that is constructed from 39 variables covering physical and cognitive function, disease conditions, psychological well-being, and other health dimensions. Survival analysis is conducted to examine how frailty is associated with subsequent mortality at an average follow-up length of 3.7 years (2.6 years for deceased persons died in 2002–2011 and 7.6 years for survived persons at the 2011 wave). Logistic regressions are applied to examine how frailty is associated with subsequent physical and cognitive functions, disease conditions, and self-rated health with an average follow-up length of 3.0 years.

**Results:**

The study reveals that centenarians are frailer than younger elders. The DI scores increase from less than 0.1 at ages 65–79 to over 0.30 in centenarians. Women are frailer than men at all ages. However, there is a great variation in frailty among all age groups. We also find that each additional increase of 0.01 score of the DI is associated with 1.6 % higher mortality risk (95 % CI: 1.014–1.018) in female centenarians and 1.4 % higher mortality risk (95 % CI: 1.010–1.018) in male centenarians, although these associations are weaker than those in other three younger age groups.

**Conclusions:**

Frailty still plays an important role in determining subsequent health outcomes and mortality in centenarians.

## Background

Research on frailty has gained increasing attention in aging studies and geriatric clinical practice in the past three decades [1, 2]. Although there is still a debate on how to define and measure frailty, there is a general agreement in the field that frailty of the later life reflects the functional decline of multiple physiological reserves that limits an elder’s ability to respond to external stressors [3–6]. Frailty is more than a health outcome out of some specific diseases or disabilities, but rather a systemic manifestation of physical and cognitive deficits that accumulate over the life course [5–8]. Frailty varies within and between individuals. Researchers have revealed that for an elder, the status of frailty could be dynamic, improved or deteriorated at different time periods [9, 10]; meanwhile it is evidenced that a substantial heterogeneity of frailty exists among individuals of old ages, as the density distribution of frailty varies greatly for elders at different ages [4, 11].

To date, there are two models to measure frailty: one is the phenotypic approach and the other is the cumulative approach [1, 4, 5]. The former defines frailty based on a set of manifest items, such as weight loss, exhaustion, weakness, slowness, or low physical activity, and takes the appearance of any three conditions as an indication of frailty [3]; whereas the latter adopts a cumulative deficit index (DI) or frailty index (FI), which quantifies an individual’s proportion of deficits in a cumulative summation over a variety of psychological, physiological, and functional conditions [7, 10]. In comparison with the phenotypic approach, the DI emphasizes the aggregate (or systemic) deterioration in psychophysiological performance [7] rather than focusing on the presence of a specific set of conditions. Numerous studies have shown that the DI is a robust predictor for health and health-related outcomes in later life such as dependent living, falls, comorbidity, disability, health change, mortality, and health care utilization, net of various confounders; and this important index has been applied as an effective tool among geriatricians, clinicians, and other practitioners for public health monitoring and intervention [2, 4, 5, 11, 12].

Frailty in centenarians has recently arisen as one of the frontline topics in the field of frailty studies [4, 13]. With the extraordinary longevity, centenarians make up a special segment of the elderly population. Studies on this group of people have been underscored in gerontology and geriatrics because of the rapidly booming size of centenarians in the world population for the recent decades [14–16]. It may not be appropriate to take it for granted that centenarians necessarily follow a general trajectory of health decline at old ages, as this group has been through significant mortality selection. In fact, some scholars argue that centenarians could be relatively healthy [17, 18]; however, more studies have refuted that centenarians tend to be the most vulnerable and sickest in terms of physical and physiological function, and other reserve capacities [19, 20]. Regardless of the debate as above, researchers tend to acknowledge that a large variation exists among centenarians in physical/cognitive functions, disease conditions as well as psychological well-being [17, 19, 21, 22]. Due to these characteristics of centenarians, to examine the prevalence and health-predictive power of frailty is warranted for this special age group.

Are centenarians necessarily frailer than younger elders? How effectively frailty could predict health outcomes in this oldest age group? To our knowledge, almost no studies in the current literature have ever attempted to address these questions, which is mainly due to data unavailability. This study chooses the largest centenarian samples in the contemporary world from the 2002, 2005, 2008, and the 2011 waves of the Chinese Longitudinal Healthy Longevity Survey (CLHLS) to investigate frailty among Chinese centenarians. Following a cumulative deficit approach, this study adopts the measurement of the DI to fulfill two specific research goals: 1) to describe the prevalence of frailty in Chinese centenarians in comparison with younger elders; and 2) to examine frailty as a predictor of subsequent mortality and health outcomes among centenarians in comparison with younger elders.

## Methods

### Study sample

**Fig. 1:**
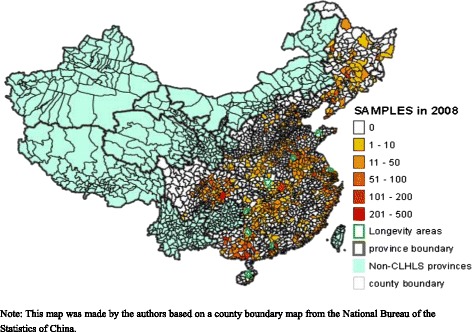
Spatial distribution of the respondents in the 2008 wave

The dataset comes from the 2002, 2005, 2008, and 2011 waves of the ongoing Chinese Longitudinal Healthy Longevity Survey (CLHLS). The first two waves of the CLHLS in 1998 and 2000 are dropped because some variables to be used in construction of the DI are not available. One purpose of each wave of the CLHLS is to interview all centenarians in a randomly selected half of the counties/cities in 22 Han-ethnicity dominant provinces in Mainland China. The 22 provinces cover about 82 % of the total population in China in 2010. Age of each centenarian in the CLHLS is validated from various sources available, including birth certificate, genealogical documents, household booklet, and ages of their children and siblings. For each centenarian interviewed, roughly one nearby respondent from each of three age groups (65–79, 80–89, and 90–99) with predesignated age and sex is randomly chosen to be interviewed based on a random code assigned to the centenarian. The nearby respondent could come from a neighboring village or town, depending on availability of the person with predesignated age and sex. All information is obtained through in-home interviews. Starting in the 2008 wave, in-depth interviews were conducted in the seven longevity areas (the number increased to eight in the 2011 wave and the 2014 wave). Figure 1 presents the spatial distribution of the sample in the 2008 wave. Detailed sampling procedures can be found in elsewhere [23]. Systematic assessments of the accuracy of age reporting, the randomness of attrition, and the reliability, validity, and consistency of numerous measures show that the data quality of the CLHLS is high [23].

For robustness of the results, we pool the 2002, 2005, and 2008 waves together to estimate the prevalence of frailty and to examine how the DI of the respondents at these three waves are associated with subsequent mortality and health outcomes in 2005, 2008 and 2011 waves, respectively. As we focus on mortality and health outcomes at subsequent waves, we only include those respondents who have at least one follow-up in 2005, 2008, or 2011. Overall, about 7500 respondents do not have any follow-up, of which 1430 are centenarian. In sum, for the mortality analysis, it has a total number of 4434 valid centenarians with 3557 women and 877 men, who make up 6541 observations with 5188 from women and 1353 from men. In three comparison groups with ages 65–99, the corresponding number for the total valid respondents is 14,051 with 7309 women and 6742 men, contributing 27,619 observations with 14,343 from women and 13,276 from men over the study period. In the health outcome analyses, only those who were healthy at the 2002, 2005, or 2008 wave were included. The valid number of sample thus varies by specific health outcome variable.

### Ethics approval

Duke University Medical Health System’s Institutional Review Board (IRB), the National Bureau of Statistics of China, and the Ethical Committee of Social Science Division of Peking University reviewed and approved ethics for this study. A written consent is obtained from each of all CLHLS participants except some rare cases when participants who are not able read or write. In these occasions, a consent form is read by the interviewer and signed by a witness.

The CLHLS datasets are publicly available at http://centerforaging.duke.edu/data-downloads. Researchers can obtain the datasets after sending a data user agreement to the CLHLS team.

### Measurements

#### Cumulative Deficit Index (DI)

Following the established research [4, 5, 24], we generate the DI for each elder with a score ranging from 0 to 1, which is measured by unweighted counts of the number of deficits divided by the total number of possible deficits (40 deficits from total 39 indicators). These 39 indicators include self-reported health status, cognitive functioning, disabilities in activities of daily living (ADL) and instrumental ADL, auditory and visual ability, depression, heart rhythm, chronic conditions (e.g., stroke, diabetes, heart diseases, lung diseases, etc.), and serious illness measured by being hospitalized or bedridden. If the number of serious illness is more than one in the past 2 years, an additional deficit score is added. More details about the measurement of the DI used in the present study can be found in the work of Gu and colleagues [4].

#### Mortality risk

Mortality risk (or hazard risk) is the dependent variable in survival analyses, which is measured by survival status and duration of exposure to death. The survival status is measured by whether a respondent interviewed in the 2002, 2005, or 2008 wave died or survived at the 2011 wave. The exposure duration for a survivor is measured by number of days from the interview date in the wave of 2002, 2005, or 2008 to the interview date in 2011. For those who died before the 2011 wave, the exposure time is measured by the time interval between date at death and the date at the interview when a respondent was first interviewed in either 2002, 2005, or 2008. The date at death was collected from officially issued death certificates whenever available, otherwise the next-of-kin and local residential committees were consulted when a death certificate was not available. The average of follow-up length from all samples used in this set of analysis is 3.7 years, with 2.6 years for deceased persons who died in the period of 2002–2011 and 7.6 years for survived persons at the 2011 wave who recruited in either 2002, 2005, or 2008. The data quality of mortality in the CLHLS has been proved to be high [23].

#### Health outcomes

Physical function, cognitive function, chronic disease conditions, self-rated health, and self-rated life satisfaction are five health outcomes used for analysis of the associations between the DI and health outcomes. Cognitive function is measured by a Chinese version of the Mini-mental Status Examination (MMSE) that includes six domains of cognition (orientation, reaction, calculation, short memory, naming, and language) with a total score of 30: a centenarian is considered as cognitively unimpaired if his or her MMSE score is 24 or over [23]. Given a low level of educational attainment of most elderly Chinese, alternative criteria are applied to respondents with different levels of education to test the sensitivity of different cut-off points for defining of cognitive impairment, and very similar results are produced (not shown but available upon request). The Chinese version of MMSE used in the CLHLS is culturally translated from the internationally standard version of the MMSE questionnaire [25]. Its validity and reliability are carefully tested in pilot surveys and are verified in each wave of the CLHLS [23, 26]. Physical function in terms of ADL is measured by whether a respondent needs any assistance in performing six daily activities, namely bathing, dressing, indoor transferring, toileting, eating, and continence. A respondent is considered as ADL independent if he or she did not need any assistance in performing all six tasks at the time of the surveys. The CLHLS adopts a list of twenty diseases (e.g., heart diseases, stroke, diabetes, hypertension, cancers, cataracts, Parkinson’s disease) to measure comorbidity; an individual is considered as having no chronic conditions if he or she did not self-report any of these twenty disease conditions at the time of the surveys. About 95 % of these self-reported diseases are confirmed by a physician or medical professional. The prevalence of each major disease is comparable to other nationwide surveys [23]. Self-rated health and self-rated life satisfaction are classified as good/very good versus others. We have also tested other categorizations for these two variables, and the findings are the same.

#### Covariates

To obtain the robust results, we follow a common practice in the literature [4, 27] to control for several sets of covariates, which include demographics, socioeconomic status, and health practice. Demographics includes age, residence (urban vs. rural), ethnicity (Han vs. non-Han), and coresidence with children (yes vs. no). Sex is not considered as a covariate since all analyses are stratified by sex. Socioeconomic status includes years of schooling (0, 1–6, and 7+), lifetime primary occupation (professional/administration vs. others), economic independence (having a retirement wage/pension and/or own earnings vs. no), and family economic condition in comparison with others (rich/very rich vs. others). Health practice is measured by currently smoking (yes vs. no), currently consuming alcohol (yes vs. no), and regularly doing exercise at present (yes vs. no).

#### Analytical strategy

The analyses involve two sets of methods and are always stratified by sex and age group with centenarians as the study group and three younger age groups 65–79, 80–89, and 90–99 as comparison. The first set of method is survival analyses, which examine whether the DI is associated with subsequent mortality risks among centenarians. Such associations are next compared with those of other age groups of 65–79, 80–89, and 90–99. The Weibull parametric survival function is applied because some variables violate the proportionality assumption of the Cox proportional hazard model. To improve its robustness, we control for demographics, socioeconomic status, and health practice. Except for sex, education, primary life occupation, all other variables are considered as time-varying variables. In other words, all longitudinal information of related variables at each follow-up wave is used in the analyses.

The second set of method is logistic regression analyses, which investigate how the DI is associated with each of five major health outcomes at the subsequent wave. The average lengthen of follow-up is 3.0 years. In this set of analyses, in order to capture changes of health at follow-up, only those who are cognitively unimpaired, ADL independent, free of chronic disease conditions, good self-rated health, or good self-rated life satisfaction at baseline waves are included. In these logistics regression models, demographics, socioeconomic status, and health practice are controlled for.

All analyses are performed using Stata version 13.1. As the sample weight of the CLHLS is purely based on the distribution of single year of age, sex, and urban–rural residence of population in the survey area and these three variables are either stratified (i.e., sex) or controlled for in the models, all regression analyses are not weighted [4]. Supplementary analyses confirm that the results in regressions from the weighted sample are very close to those from the unweighted. However, in description of the DI score, the sample weight is applied.

## Results

**Fig. 2:**
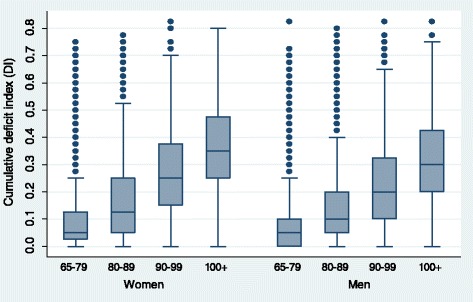
Distribution of median and quartile percentile of cumulative deficit index by age group and sex, CLHLS 2002–2008, pooled, weighted

**Fig. 3:**
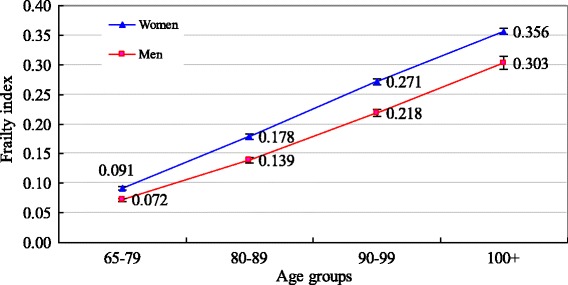
Mean scores of DI and their 95 % CIs by age group and sex, CLHLS 2002–2008, pooled, weighted

**Fig. 4:**
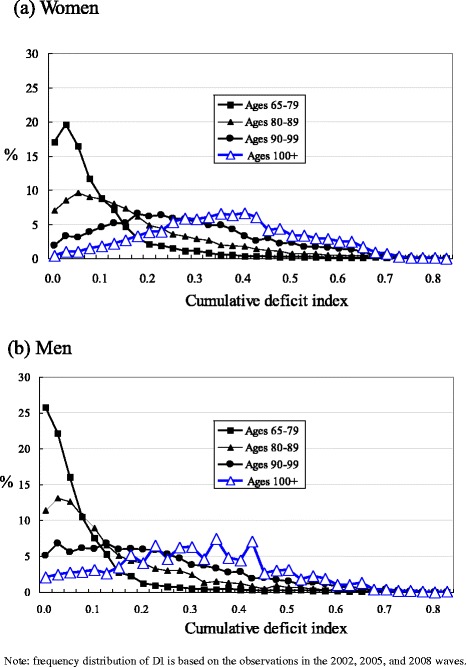
Distribution of the cumulative deficit index by age group and sex, CLHLS 2002–2008, pooled, weighted

**Fig. 5:**
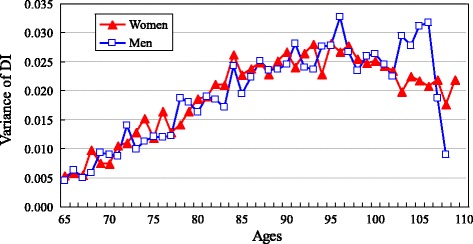
Variance of DI by age and sex, CLHLS 2002–2008, pooled, weighted

The sample characteristics are presented in Table 1. Figure 2 uses box plots to present the median and quartile percentiles of the DI scores for centenarians by sex in comparison with other three age groups in the pooled dataset. Figure 3 shows the mean and 95 % confidence intervals of the DI scores by sex for four age groups. Results in Figs. 2 and 3 indicate that centenarians tend to have a higher DI score, with 0.356 for women and 0.303 for men. The mean DI scores of other three comparison groups for women are 0.091 for ages 65–79, 0.178 for ages 80–89, and 0.271 for ages 90–99, respectively. The corresponding figures of for men are 0.072, 0.139, and 0.218, respectively. Figure 4 reports the density distribution of the DI scores for centenarians and other three age groups by sex in the pooled dataset. This distribution is very similar to those in Gu and colleagues [4], indicating that in comparison with younger age groups, most centenarians have a higher DI score. Figure 5 shows that the variance of the DI scores increases with age up to ages 95–99 and then slightly declines in centenarians with great fluctuations, regardless of sex This indicates that there exists a large variation of the DI scores in centenarians.

**Table 1:** Sample description of the CLHLS, 2002–2008, pooled, unweighted

	Centenarians		Ages 65–99	
Variables	Women	Men	Women	Men
# of respondents (observations)	3,557 (5,188)	877 (1,353)	7,309 (14,343)	6,742 (13,276)
Mean DI score (*100)	36.9	30.8	18.1	13.3
*Health outcomes*				
% ADL independent	42.6	52.0	80.9	87.4
% Cognitively unimpaired	18.3	33.5	60.7	76.8
% Having no chronic diseases	44.4	44.6	41.3	43.1
% Self-rated good health	35.6	43.7	45.9	50.8
% Self-rated good life satisfaction	46.2	52.5	56.2	56.2
% Death from 2002 to 2011/12	79.3	82.2	36.0	35.8
*Covariates*				
Mean age	101.4	101.3	82.7	80.8
% Urban	36.8	43.7	36.4	37.8
% Han ethnicity	94.6	93.3	93.8	94.3
% Coresidence with children	87.5	80.2	65.3	55.2
% 0 years of schooling	92.5	57.0	79.4	36.4
% 1–6 years of schooling	6.2	34.4	16.5	45.9
% 7+ years of schooling	1.3	8.6	4.1	17.7
% White collar job	0.8	8.2	3.2	13.2
% Economic independence	2.4	23.7	23.8	45.6
% Good family economic condition	13.6	16.1	14.4	16.5
% Smoking at present	4.9	20.9	7.4	38.4
% Alcoholic consuming at present	12.5	24.3	9.3	33.9
% Doing regular exercise at present	13.9	26.7	24.8	36.0

*Note:* (1) DI refers to the cumulative deficit index. (2) The samples and observations only include those who were aged 65–105 at their first interviews in the 2002, 2005 and 2008 waves of the CLHLS and had at least one follow-up interview. (3) Except for the proportion of death, which is calculated based on the whole study period, five health outcomes are measured at either 2002, 2005, or 2008 wave of the CLHLS

**Fig. 6:**
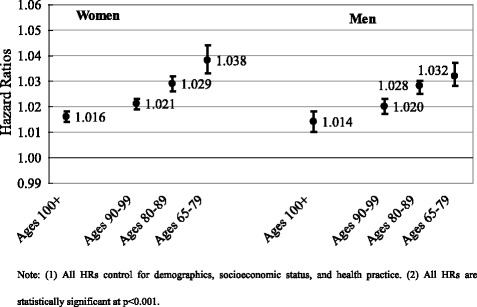
Mortality hazard ratios of frailty index score (*100) in centenarians in comparison with non-centenarians by sex, the CLHLS 2002–2011, pooled, unweighted

Figure 6 reports hazard ratios of mortality, adjusting for demographics, socioeconomic status, and health practice. In centenarians, each additional 0.01 increase in the DI score is associated with 1.6 % and 1.4 % higher hazard ratios of mortality in women and men, respectively, lower than those in other age groups. Although Fig. 6 reveals a diminishing association between the DI score and mortality risk over ages, it is clear that the DI still maters to mortality in centenarians.

Table 2 presents sex-specific odds ratios of subsequently being in a poor health condition for each additional 0.01 increase in the DI score, in presence of controls such as demographics, socioeconomic status, and health practice. The results clearly show that in centenarians, a higher DI score is associated with greater risks of being disabled, cognitively impaired, poor in self-rated health, and poor in self-rated life satisfaction. The increased odds ratios are relatively larger in men than in women. Such negative associations are also observed in other age groups. There are a couple of exceptions, however. For example, the association between the DI scores and chronic diseases are not significant at *p* < 0.05 for centenarians, and this is also the case for women in age groups of 80–89 and 90–99.

**Table 2:** Odds ratios of being in a poor health condition at the subsequent wave for the DI by age and sex, CLHLS 2002–2011, pooled, unweighted

	Women	Men
From ADL independent to ADL disable		
Ages 100+ (291/125)	1.035 (1.021–1.049)***	1.048 (1.021–1.077)***
Ages 90–99 (743/679)	1.036 (1.026–1.045)***	1.036 (1.025–1.048)***
Ages 80–89 (1480/1633)	1.045 (1.036–1.054)***	1.043 (1.033–1.053)***
Ages 65–79 (3055/3390)	1.058 (1.045–1.070)***	1.066 (1.053–1.080)***
From cognitively unimpaired to impaired		
Ages 100+ (550/159)	1.024 (1.003–1.046)*	1.039 (1.011–1.069)**
Ages 90–99 (1128/833)	1.013 (1.008–1.031)**	1.035 (1.022–1.048)***
Ages 80–89 (1838/1794)	1.011 (1.003–1.019)**	1.026 (1.017–1.035)***
Ages 65–79 (3351/3471)	1.020 (1.011–1.030)***	1.035 (1.025–1.046) ***
From no chronic diseases to having 1+ chronic diseases		
Ages 100+ (462/113)	1.007 (0.995–1.018)	1.024 (0.998–1.050) +
Ages 90–99 (649/487)	1.009 (1.000–1.019) +	1.019 (1.005–1.032)**
Ages 80–89 (848/808)	1.010 (0.999–1.021) +	1.021 (1.007–1.034)**
Ages 65–79 (1347/1481)	1.022 (1.001–1.040)*	1.027 (1.008–1.047)**
From good self-rated health to poor self-rated health		
Ages 100+ (465/144)	1.029 (1.018–1.039)***	1.051 (1.026–1.077)***
Ages 90–99 (749/593)	1.022 (1.014–1.030)***	1.027 (1.016–1.038)***
Ages 80–89 (1105/1083)	1.033 (1.025–1.041)***	1.039 (1.029–1.049)***
Ages 65–79 (1738/1959)	1.043 (1.033–1.054)***	1.058 (1.047–1.070)***
From good self-rated life satisfaction to poor self-rated life satisfaction		
Ages 100+ (582/151)	1.019 (1.007–1.030)**	1.051 (1.023–1.080)***
Ages 90–99 (931/652)	1.016 (1.001–1.025)***	1.011 (0.999–1.021) +
Ages 80–89 (1315/1218)	1.011 (1.003–1.018)**	1.005 (0.996–1.014)
Ages 65–79 (1979/2073)	1.016 (1.006–1.025)**	1.022 (1.012–1.032)***

*Note:* (1) Odds ratios in this table are adjusted for demographics, socioeconomic status, and health practice. (2) numbers in parentheses at the first column are number of observations of women and men included in the regression models, respectively. (3) + p < 0.1, *p < 0.05, **p < 0.01, ***p < 0.001

## Discussion

Frailty has become one of the key topics in the aging study since the 1980s [28, 29]. Due to the capacity of reflecting accumulative health risks in old ages, this measurement holds important values in research and practice, especially for the public health system to face the unprecedented trend of population aging [30, 31]. However the current literature has not adequately studied this important measurement in centenarians [13]. In particular, the prevalence and mortality/health predictive power of frailty are yet unexplored for this group of population with extraordinary longevity.

This study makes an initial effort to fulfill such a research gap and it has some important strengths. First, unlike most centenarian studies which hold relatively small sample sizes [5, 8], we have employed the world’s largest sample of centenarians, as guarantees the robustness of the analysis. Second, our study samples come from the mainland China, which has the world’s largest oldest-old population since the 1990s and up until the end of the 21st century [32]. In comparison with the Western literature, research on frailty is still scarce in developing countries. With regard to China, there is an urgent call to better understand health-related issues such as frailty, not only because the Chinese population is aging rapidly, but also because the Chinese eldercare system is experiencing profound changes [33].

This study provides robust evidence showing that centenarians are frailer in average than their younger elderly counterparts. The distribution of the DI scores over ages in this study is almost similar to those reported in one previous study on China [4]. The mean DI score over younger ages (before age 100) as reported in the study is also similar to those from other populations, such as in the U.S. [5, 7] and in a pooled sample from a few developed countries [10].

Although centenarians appear to be the frailest among all elders, this study reveals a substantial heterogeneity within this group, at least greater than those below age 90. The DI score distribution of centenarians as observed in the study well echoes the mathematical models of frailty as proposed by Rockwood and colleagues [6, 34, 35], who argue that the distribution of the frailty index tends to change from a gamma distribution to approximate a normal one with advance of age. Such a distributional change has been justified from a biological perspective. That is, aging could be considered as a consequence of system redundancy: with deficits being accumulated through the life course, the exhaustion of redundancy at the ending years will lead to the emergence of a normal distribution in frailty [36]. This study provides empirical evidence to supporting the models proposed by Rockwood and colleagues. With a couple of exceptions [4, 37], previous work has never examined frailty distribution for centenarians.

Furthermore, we find that women are frailer than their male counterparts, and this sex disparity gets smaller at younger ages. Such a finding is in line with what has been found in other populations [2, 5, 7, 10, 38]. We speculate that the higher level of frailty in women might be due to a combination of higher incidence, longer durations (i.e., low recovery), and lower severity of illnesses [39]. The literature of aging studies has long recognized a gender paradox in health and mortality, and it is argued that gender differences in genetic and acquired risks, immune system responses, hormones, disease patterns and prevention, and health-reporting behaviors may explain the poorer health and lower mortality of women than men [40]. Due to unavailability of data and research on frailty in centenarians, we are not able to make comparisons between China and other countries to examine whether the sex difference in the DI scores persists. However, two recent studies focusing on near-centenarians and centenarians show that women are frailer than men in terms of phenotypic frailty [41] and accumulative deficit index [13]. More studies are clearly needed to further verify the gender difference.

One critical finding in this study is that the DI score is still a valid predictor of mortality among centenarians. Previous studies have argued that the DI tends to have a better predictive ability of mortality than the phenotype measurement [42], and according to the review by Bouillon et al. [1], using the DI or the frailty index, the odds ratios of mortality range from 1.57 to 10.53 for frail individuals relative to non-frail individuals. However, to our knowledge, how the DI could be predictive of mortality among centenarians has not been investigated. Our results for the first time show that the DI still maintains predictive of mortality in this very old aged population, although its power is not as strong as those in younger cohorts. Such a finding is reasonable. On the one hand, the DI reflects a universal feature of the aging process, in which physical, psychological, and social deficits accumulate over time; and its predictive power over adverse health outcomes is independent of chronological age [5]. Thus the basic principle of the DI in health prediction that “people with more things wrong are more like to suffer an adverse event” [6:723] should be applied to all human beings, regardless of how old the chronological age is. On the other hand, at the extremely old ages such as those of centenarians, the heterogeneity of frailty is often greater than that of younger ages due to mortality deceleration among centenarians [43, 44], therefore the accruement of each deficit in the DI may gradually lose its power in mortality prediction.

Besides mortality, this study also finds out that the DI is a valid measure in predicting important health outcomes in centenarians such as cognitive impairment, ADL disability, poor self-reported health, and poor self-reported life satisfaction. The only exception is comorbidity, which is likely due to the fact that comorbidity is prevalent at old ages. Moreover, the underreport of diseases and conditions in old ages, which is quite likely in China, especially its rural areas, could be another possible explanation. We consider that some Chinese elderly people possibly do not know that they have diseases due to the underdeveloped local health service system and poor health literacy. In addition, the preference on traditional Chinese medicine may also be a barrier for potential patients to seek help from a physician [45, 46]. Nevertheless, we think the data quality of disease reporting in the CLHLS may not bring in major biases in the estimation in that the prevalence of major diseases reported in the CLHLS is indeed comparable to a few other nationwide surveys in China [23]. It is worth noting that frailty loses its predictive power on comorbidity in women of 80–89 and 90–99. More research is needed to shed the lights on such issues with further improving data accuracy of chronic conditions in developing countries such as China.

Our study has some important implications in both public health and clinical practices. With the life expectancy being prolonged, the current challenge of the world population is to achieve healthier survival at old ages. To identify, avoid, delay, and reverse frailty at old ages could make up an effective way of preventing adverse health outcomes and improving the quality of life of older persons. Nevertheless, to fully accommodate frailty in the clinical practices still requires further transformation of the current medical system, which has been long institutionalized around single-system illness [34]. Meanwhile, there is still a long way for the public health system incorporating the notion of frailty to monitor and manage the population health beyond merely targeting specific diseases and conditions, although the gain appear to be substantial [47]. Through confirming the validity of the DI in predicting negative health outcomes in centenarians, our study supports the application of this important measurement for the full spectrum of old ages. In this sense, this study contributes empirical evidence to supporting the use of the frailty measurement in policy and clinical practices, which could enhance health agencies to better educate people to prepare for the catastrophic negative events in later life. Moreover, our finding on the great heterogeneity of frailty among centenarians deserves special attentions. As centenarians are often taken as a symbol of successful aging, our findings urge future studies to explore factors responsible for the substantial health disparity in this very old age group. In particular, the non-frail centenarians may provide a key for us to understand how to achieve healthy longevity.

Our study has some limitations. For example, we only tested one specific construct of the DI, which is based on 39 indicators. The health predictive power may vary if different variables are used in constructing the DI. Thus more studies are definitely warranted to examine the validity of the DI in centenarians by using different measures. However, from our point of view, it is quite unlikely for the other constructs of the DI to have outcomes against the findings of the present study because as long as the inventory of variables includes main domains of the DI, such as disability, functional limitation, cognitive impairment, psychological distress, and chronic diseases, the results are mostly likely to be similar [4].

## Conclusions

Centenarians appear to be the frailest among all elders, yet within this group there exists a large heterogeneity in frailty. Women centenarians are frailer than their male counterparts, and the sex disparity is smaller in younger age groups. For centenarians, frailty when meaured by the DI score still plays an important role in affecting mortality, cognitive impairment, ADL disability, poor self-reported health, and poor self-reported life satisfaction, although the predictive powers of the DI are not as strong as those in younger cohorts. Our findings suggest that to identify, avoid, delay, and reverse frailty at old ages could be an effective way to prevent adverse health outcomes and to improve the quality of life of older persons, and to understand how elders maintain not frail may provide a key to understanding how to achieve healthy longevity.

## Abbreviations

ADL: Activities of daily living
CLHLS: Chinese Longitudinal Healthy Longevity Survey
DI: Cumulative deficit index
FI: Frailty index
MMSE: Mini-mental status examination
